# Autophagy Induced by ROS Aggravates Testis Oxidative Damage in Diabetes via Breaking the Feedforward Loop Linking p62 and Nrf2

**DOI:** 10.1155/2020/7156579

**Published:** 2020-05-18

**Authors:** Yuanyuan Tian, Wei Song, Dongsheng Xu, Xiao Chen, Xiaojiao Li, Yuguang Zhao

**Affiliations:** ^1^Cancer Center, The First Hospital of Jilin University, Changchun, Jilin 130021, China; ^2^Phase I Clinical Trials Unit, The First Hospital of Jilin University, Changchun, Jilin 130021, China

## Abstract

Testicular dysfunction due to hyperglycemia is the main cause of infertility in diabetic men. Over the years, in order to solve this growing problem, a lot of research has been done and a variety of treatments have been created, but so far, there is no safe, effective, and practical method to prevent male infertility caused by diabetes. In this review, we emphasize the male infertility mechanism caused by diabetes from the effects of oxidative stress and autophagy on the function of testes via the PI3K/Akt/mTOR signaling pathway, and we highlight that oxidative stress-induced autophagy breaks the feedforward loop linking Nrf2 and p62 and promotes oxidative damage in diabetic testes.

## 1. Introduction

As a multifactorial disease characterized by hyperglycemia, the incidence of diabetes has been increasing in the past decades [1]. According to the latest data, 463 million adults currently have diabetes. If adequate action is not taken to address the epidemic, 578 million people will have diabetes by 2030. By 2045, that number will jump to a staggering 700 million [2]. It is well known that diabetes can negatively affect the fertility of women and men, and studies have shown that diabetes can cause cellular abnormalities in reproductive organs [3–6]. Testicular dysfunction is a major complication of diabetic patients, especially those at reproductive age, and its incidence is increasing worldwide [7–9]. In male reproductive organs, testes are most vulnerable to hyperglycemia [9]. Animal studies using the diabetic rodent models have shown that diabetes can damage the epididymis of rodents, influence the quality of semen, and thus impair reproductive function [10–13]. Previous clinical studies have confirmed that sperm count, motility, and morphology in diabetic patients are significantly worse compared to the control group [14–16]. In addition, previous studies have demonstrated that diabetic rats have reduced testicular weight [17], abnormal tissue structure of seminiferous epithelium, vacuolization of Sertoli cells (SCs) [18], and disruption of the blood-testis barrier (BTB) [19]. Therefore, it is of great significance to study the mechanism of testicular dysfunction in diabetic patients and find an effective way to prevent male infertility.

In the pathogenesis of male infertility in diabetes, reactive oxygen species (ROS) play a vital role [20]. And under a variety of pathological conditions, the relative excessive accumulation of ROS can induce autophagy [21–24]. In previous animal model studies, it has also been confirmed that in the testis, excessive production of ROS can induce autophagy [25–27]. Therefore, in this review, we speculate that in diabetes, excessive production of ROS can induce autophagy in the testis. A series of studies have confirmed that abnormal autophagy can cause abnormalities in the complex and highly ordered sperm cell differentiation process, such as acrosome biogenesis and sperm differentiation defects [28, 29], decreased serum testosterone levels [30], and SC apoptosis and BTB damage [26, 31]. The phosphatidylinositol 3-kinase (PI3K)/protein kinase B (Akt)/mammalian target of rapamycin (mTOR) signaling pathway is a target of oxidative stress [32]. Furthermore, the PI3K/Akt/mTOR signaling pathway is one of the most vital regulators of autophagy and its activation promotes spermatogenesis [33–35]. In this review, we mainly elaborate that autophagy induced by oxidative stress via the PI3K/Akt/mTOR signaling pathway accelerates the oxidative stress in the testis, and we highlight that autophagy induced by oxidative stress breaks the feedforward loop linking Nrf2 and p62 and aggravates oxidative damage in diabetic testes.

## 2. Diabetes and Male Infertility

Diabetes mellitus (DM) is a multifactorial disease characterized by hyperglycemia. In the past few decades, a lot of work has been done and a variety of treatments have been developed to address this growing problem; however, even today, the prevalence and incidence of diabetes continues to rise sharply. And the incidence of type 1 diabetes (T1D) has been increasing worldwide [36]. At the same time, as the incidence is increasing, the age of onset is also getting younger. According to studies evaluating temporal trends, the incidence of childhood-onset T1D has increased around the world, with an average relative growth of 3%-4% per calendar year [37]. This has many implications for the risk of complications. The increased incidence of diabetes in young people is of great concern because it may affect the reproductive function of more men during their active reproductive age.

## 3. Hyperglycemia Inducing the Production of ROS

**Figure 1:**
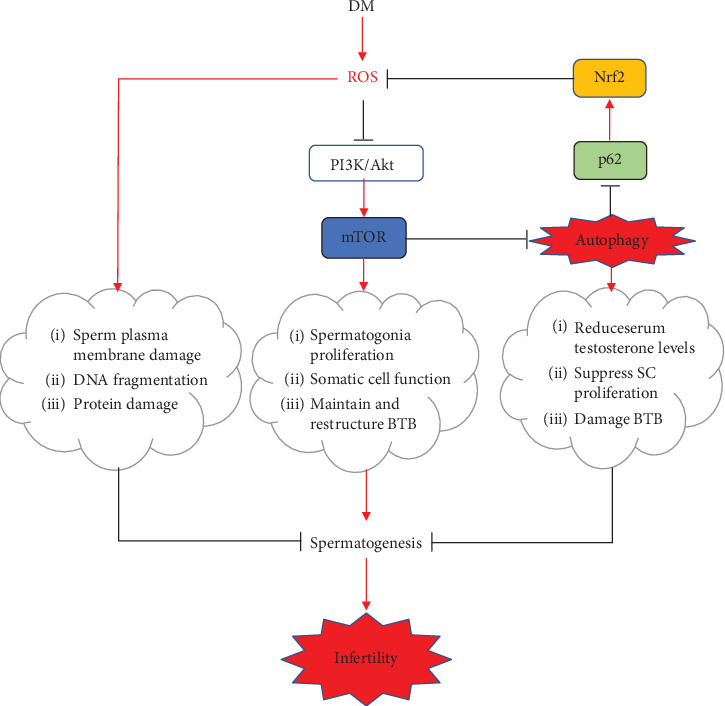
Mechanistic illustrations. The increasing formation of ROS in DM can directly cause damage to spermatogenesis via attacking the fluidity of the plasma membrane lipids, protein modifications, and integrity of DNA. And ROS also can induce autophagy by inhibiting mTOR through the PI3K/Akt signaling pathway. Autophagy accelerates the degradation of p62, and then, the Nrf2 activation is suppressed and the oxidative damage is aggravated. Autophagy also directly causes damage to spermatogenesis via reducing serum testosterone levels, suppressing SC proliferation, and damaging BTB. Moreover, mTOR has distinct effects on spermatogenesis via promoting spermatogonia proliferation, maintaining somatic cell function, and restructuring BTB. As a result, the oxidative damage in diabetic testes is further enhanced, thereby promoting the occurrence of infertility.

Diabetes is characterized by a hyperglycemic state, and the elevating level of oxidative stress directly induced by hyperglycemia plays a crucial role in male infertility [38] (Figure 1). Hyperglycemia has been shown to cause oxidative stress through hydroxyl radicals generated by the autoxidation of glucose [39, 40]. Previous investigations have demonstrated that accumulation of advanced glycation end products (AGEs) produced by nonenzymatic reactions between sugar and amino groups of proteins under hyperglycemic conditions and their receptors (RAGE) plays a crucial role in the development of diabetes-induced complications, including the pathogenesis of diabetes-induced male reproductive damage, which activates oxidative stress and increases the generation of ROS [19, 41, 42]. Through semiquantitative analysis of immunostaining of sperm from the diabetic and nondiabetic males, Mallidis et al. also found that in diabetic male sperm samples, the majority of sperm (>60%) expressed RAGE, which is approximately three times greater than that seen in samples from nondiabetic men [43]. Therefore, in patients with diabetes, excessive hydroxyl radicals and AGEs induced by hyperglycemia can further increase the production of ROS, leading to an increase in oxidative stress and aggravation of sperm loss.

## 4. The Role of ROS in Testicular Functions

As one of the messengers that affect sperm function during the process of sperm from the testis to the oocyte, low levels of ROS can regulate sperm function, promote sperm capacitation, and regulate sperm maturation [44]. Numerous studies have shown that low levels of ROS are essential in the process of obtaining fertilization in sperm. Aitken et al. first proposed the idea that low levels of ROS can regulate the physiological functions of sperm and found that the ability of sperm to bind the zona pellucida can be enhanced by low levels of ROS [45]. Adding low concentration of hydrogen peroxide (H_2_O_2_) can stimulate sperm capacitation, overactivation, acrosome reaction, and oocyte fusion, which has also been confirmed by studies [46, 47]. ROS other than H_2_O_2_ such as nitric oxide and superoxide anion have also been shown to promote sperm capacitation and acrosome reaction [48, 49]. However, human sperm is particularly vulnerable to oxidative damage, and an imbalance between the production of ROS and the antioxidant capacity of sperm will lead to male infertility [50–52]. It has been accepted that spermatozoa are vulnerable to oxidative damage because their plasma membranes contain large amounts of polyunsaturated fatty acids (PUFA) and have low concentrations of scavenging enzymes in their cytoplasm [13, 53, 54]. When levels are excessive, ROS attack PUFA in the sperm plasma membrane, leading to lipid peroxidation [55]. A large number of studies have shown that long-term hyperglycemia increases the production of ROS in testicular tissues in patients with diabetes and plays a key role in male testicular dysfunction [6, 56–58] (Figure 1).

### 4.1. Imbalance of Oxidation and Antioxidation in Testes Leads to Male Infertility

Among diabetics, oxidative imbalance is a key hallmark in testes. Hyperglycemia induces the germ cells and somatic cells in the testes to produce excessive ROS [19, 59], which overwhelms the endogenous ROS scavenging systems [60, 61]. Oxidative stress further causes damage to many macromolecules and disrupts their functions, such as lipid peroxidation, protein modifications, and DNA damage [62–64] (Figure 1). And the sperm cell membrane damage induced by oxidative stress may be one of the reasons for infertility [52, 65]. It has been proved that ROS attacks not only the fluidity of the sperm plasma membrane but also the integrity of DNA in the sperm nucleus [66]. Agarwal et al. have found that the level of apoptosis of mature sperm in infertile patients is significantly higher than that in normal donors in the control group, indicating that DNA damage caused by ROS may accelerate apoptosis of germ cells, further leading to a significant decrease in sperm count and semen quality [67]. The level of DNA oxidation in infertile men's sperm is higher than that in fertile men's sperm, which further proves that the excessive production of ROS causes damage to sperm [68]. Therefore, improving the antioxidative ability of testes may become an effective method for preventing male infertility in diabetic patients.

### 4.2. PI3K/Akt/mTOR Signaling Pathway as a Central Regulator of Spermatogenesis

The activation of PI3K and its downstream target mTOR is beneficial to protein synthesis and cell survival, which has been confirmed by previous studies. Blume et al. have confirmed that the activation of PI3K is essential for stem cell factor-induced spermatogenesis [69]. mTOR, a well-conserved Ser/Thr protein kinase, plays a key role in sensing environmental conditions and regulating cell metabolism [70]. Since the first clinical evidence showed that a 36-year-old male was infertile after taking rapamycin, an mTOR inhibitor, and his sperm analysis showed a dramatic diminution of sperm count, percentage of normal-shaped sperm heads, and sperm motility, a role for mTOR in male reproductive physiology was originally proposed [71]. Studies have verified distinct roles for mTOR in spermatogenesis [72] (Figure 1). Deutsch et al. found that testosterone secretion and sperm count decreased in patients treated with rapamycin, and sperm count and sex hormone levels improved after withdrawing rapamycin [72]. Recent studies have shown that mTOR plays a key role in testicular physiology. mTOR has two different complex forms, mTOR complex 1 (mTORC1) and mTOR complex 2 (mTORC2), which increases the complexity of studying the function of mTOR [73]. mTOR and its associated partner proteins are expressed in both the germ (particularly in spermatogonia) and somatic (Sertoli and Leydig) cells within the testis, with mTORC1 and mTORC2 being differentially present in all these cellular types [74–77]. In spermatogonia, the application of rapamycin caused a decrease in proliferation by blocking the mTOR/p70S6K (70 kDa ribosomal protein S6 kinase, a significant downstream effector of mTOR, mediating protein synthesis) pathway, which indicates the role of mTORC1 in maintaining germ cell proliferation [77]. In vivo studies have shown that rapamycin causes atrophy and vacuolation of seminiferous tubules by inhibiting mTORC1, leading to reduced sperm production, which indicates the role of mTORC1 in spermatogenesis [77]. mTORC1-deficient mice show reduced sperm motility, which indicates that mTORC1 can regulate the physiological functions of sperm during the passage of the epididymis, in addition to maintaining germ cell proliferation and spermatogenesis [76]. Studies show that mTOR can directly participate in nutritional support for spermatogenesis by controlling glucose consumption and redox balance in SCs [78]. In addition, mTOR also plays a key role in the maintenance and reorganization of BTB, which is very important for maintaining the spermatogenic epithelium circulation [79, 80]. Although mTOR has been shown to participate in many physiological processes, the role of mTOR and its inhibitors in male reproduction needs more research.

### 4.3. ROS Inducing Autophagy via Inhibiting the PI3K/Akt Signaling Pathway

Under various stress conditions, such as hyperglycemia, oxidative stress, and starvation, the PI3K/Akt/mTOR signaling pathway is a typical negative regulatory pathway for autophagy in mammalian germ cells. Studies have reported that overproduction of ROS in long-term hyperglycemic organisms significantly inhibits the PI3K/Akt signaling pathway, thus affecting cell autophagic function (Figure 1). Lin et al. found that ROS induced autophagic cell death by negatively regulating this signaling pathway [81]. Shi et al. also demonstrated that the levels of p-PI3K and p-Akt/t-Akt in diabetic testes cells were significantly downregulated, whereas after treatment with Lycium barbarum polysaccharide, a well-known antioxidant food supplement, p85-PI3K and p-Akt expression were significantly upregulated [82]. Therefore, under hyperglycemic conditions, reducing ROS production can regulate autophagy through a PI3K/Akt-dependent mechanism in testicular tissues. It has also been proved that the increase of oxidative stress inhibits the PI3K/Akt/mTOR pathway, following by the inhibition of the expression of p70S6K, leading to degeneration and malformation of sperm, and affects sperm count, motility, and function in epididymis [77, 83].

## 5. The Role of Autophagy in Male Testicular Functions

Autophagy is an intracellular lysosomal degradation pathway and plays a very important role in maintaining intracellular homeostasis [84]. The main role of autophagy is to eliminate intracellular energy resources in nutrient shortage conditions and remove cytotoxic proteins and organelles under stressful situations [85]. Previous studies have shown that autophagy plays an important role in acrosome biogenesis [29] and spermatid differentiation during spermatogenesis [28]. Moderate autophagy maintains homeostasis of organisms and was reported to play a protective role against testicular damage caused by hyperglycemia [85] and hypoxia [86]. However, a series of research studies confirmed that abnormal autophagy is pivotal for male infertility (Figure 1). Leydig cells, as an important part of the testicular stroma, are the main source of androgens [87]. Zhao et al. have shown that autophagy induced by suppressing the Akt-mTOR pathway can inhibit Leydig cells, thereby reducing serum testosterone levels [30]. SCs are essential for spermatogenesis and male fertility, and they coordinate the spermatogenesis process by providing nutrition and an environment conducive to the survival and development of germ cells [88–90]. It has been reported by Duan et al. that in SCs, the mTOR signaling pathway mediated by ROS may be the main pathway to augment autophagy, which causes the suppression of SC proliferation, thus impairing spermatogenesis and fertility [31]. The BTB consisting of tight junctions, adherens junctions, and gap junctions between adjoining SCs plays a key role in the spermatogenesis microenvironment and is a well-known premise of spermatogenesis [91–93]. Yi et al. have proved that the accumulation of autophagosome affects the integrity of BTB, which finally contributes to spermatogenesis disturbance, accumulation of damaged mitochondria, and infertility [26]. Therefore, reducing autophagy induced by ROS may become an effective method for preventing male infertility in diabetic patients.

## 6. Autophagy Aggravating the Oxidative Damage in Testes

The autophagy-related protein p62, as a scaffold protein, binds ubiquitinated substrates and aids their aggregation and degradation by macroautophagy [94]. And as a target of autophagy, p62 is constantly controlled by constitutive autophagy [95, 96] (Figure 1). Komatsu and colleagues have also shown that p62, as an endogenous protein, activates nuclear factor erythroid 2-related factor 2 (Nrf2) by competitive combination of Kelch-like ECH-associated protein 1 (Keap1), a redox-sensitive E3 ubiquitin ligase substrate adaptor, which strictly regulates the intracellular Nrf2 abundance [97, 98]. Nrf2, a key factor in the cellular antioxidant system, can respond to oxidative stress [99–101]. Under homeostatic conditions, low levels of Nrf2 are primarily maintained by Keap1-mediated proteasomal degradation [102]. Under oxidative stress conditions, Keap1 is oxidized at reactive cysteine residues, resulting in inactivation of Keap1 and stabilization of Nrf2, which then translocates into the nucleus and subsequently binds to antioxidant response elements to promote the expression of downstream cytoprotective proteins that act as scavengers for diabetes-induced free radicals [103–105]. Jain and colleagues also demonstrated that p62 creates a positive feedback loop in the Keap1-Nrf2 pathway and the loop will be broken by the autophagic degradation of p62 [106] (Figure 1). In previous studies, we have found that p62 expression was significantly decreased, Keap1 was significantly increased, and the ratio of nuclear Nrf2 to cytosolic Nrf2 was decreased in the T1D group [107]. We speculate that in the T1D group, when the autophagy is induced by ROS, p62 is degraded and the feedforward loop linking Nrf2 and p62 is broken, which directly results in a decrease in antioxidant capacity and an increase in ROS (Figure 1).

Increased Nrf2 expression can increase the antioxidant capacity of sperm in diabetic patients, which has been confirmed in previous studies. Jiang et al. have shown that sulforaphane may prevent testicular oxidative damage and apoptosis by increasing testicular Nrf2 expression under diabetic condition [108]. Pan et al. have proved that the Nrf2 knockout mice exhibited more significant diabetes-induced loss in testicular weight and sperm count, as well as increased testicular apoptotic cell death compared to wild-type mice, and have demonstrated that Nrf2 plays a critical role in ameliorating diabetic testicular damage [109]. We have also shown that resveratrol can attenuate testicular apoptosis in T1D mice via activating Nrf2 through the PI3K/Akt pathway and p62-dependent Keap1 degradation in our previous study [107] (Figure 1). Therefore, increasing the expression of Nrf2 may become an effective method for preventing male infertility.

## 7. Conclusion

As mentioned above, infertility is a common complication of diabetic men, and there is already some evidence to support the role of ROS and autophagy in the pathophysiology of male infertility caused by diabetes. In this review, we elucidate the interaction between ROS and autophagy in diabetic testes via the PI3K/Akt/mTOR signaling pathway and highlight that autophagy induced by ROS aggravates oxidative damage via breaking the feedforward loop linking Nrf2 and p62. Moreover, we have suggested that reducing the production of ROS via decreasing the serum glucose concentration may be effective to treat and prevent male infertility in diabetic patients. And we have also showed that upregulating the Nrf2-Keap1 pathway can increase the ratio of nuclear Nrf2 to cytosolic Nrf2 and enhance the transcription of antioxidant enzymes, such as superoxide dismutase, glutathione peroxidase, and catalase. Additionally, supplementation with nonenzymatic antioxidants such as resveratrol, glutathione, carnitine, pyruvate, vitamin C (ascorbic acid), and vitamin E (*α*-tocopherol) may be effective to augment the scavenging capacity of testes.

Although it has been demonstrated that ROS could promote the formation of autophagy, in turn, autophagy may contribute to aggravate oxidative damage by degrading p62; the internal molecular regulatory mechanisms between ROS and autophagy are complicated in diabetic testicular cells and still need further research.

## Abbreviations

ROS: Reactive oxygen species
SCs: Sertoli cells
BTB: Blood-testis barrier
PI3K: Phosphatidylinositol 3-kinase
Akt: Protein kinase B
mTOR: Mammalian target of rapamycin
DM: Diabetes mellitus
AGEs: Advanced glycation end products
RAGE: Receptors of advanced glycation end products
PUFA: Polyunsaturated fatty acids
H_2_O_2_: Hydrogen peroxide
LC3B: Light chain 3B
Nrf2: Nuclear factor erythroid 2-related factor 2
Keap1: Kelch-like ECH-associated protein 1.
